# HIV-1-infected cell-derived exosomes promote the growth and progression of cervical cancer

**DOI:** 10.7150/ijbs.38146

**Published:** 2019-09-07

**Authors:** Haiyu Li, Xiangbo Chi, Rong Li, Jing Ouyang, Yaokai Chen

**Affiliations:** 1 Department of Infectious Diseases, Chongqing Public Health Medical Center, Chongqing, China; 2 Departments of Department of Gastroenterology, Chongqing Public Health Medical Center, Southwest University, Chongqing, China

**Keywords:** cervical cancer, HIV-infected T-cell, exosome, miR-155-5p

## Abstract

**Background**: Women infected with HIV are more likely to have aggressive cervical cancer, and patients with HIV infection are often more severely ill than those without HIV infection. However, the underlying mechanism for the progression of cervical cancer is not yet fully understood and requires further research.

**Methods**: Exosomes were isolated from cell culture supernatants using differential ultracentrifugation. Confirmation of exosome isolation was based upon identification by electron microscopy and NanoSight particle tracking analysis of the purified fraction. The function of exosomes derived from HIV-infected T-cells in cervical cancer was determined by CCK8 and Transwell invasion assays.

**Results**: Exosomal miR-155-5p derived from HIV-infected T-cells promotes the proliferation, migration and invasion of cervical cancer cells. Furthermore, we found that HIV-infected T-cells secrete exosomal miR-155-5p that directly targets ARID2 degradation, leading to activation of the NF-κB signaling pathway. MiR-155-5p promotes cervical cancer progression by secreting proinflammatory cytokines, including IL-6 and IL-8.

**Conclusions**: In conclusion, we demonstrate that intercellular crosstalk between HIV-infected T-cells and cervical cancer is mediated by exosomes from HIV-infected T-cells that contribute to the malignant progression of cervical cancer, providing potential targets for the prevention and treatment of HIV-associated cervical cancer.

## Introduction

Women infected with HIV are more likely to have aggressive cervical cancer, and those with HIV infection at the time of consultation are often more serious cases than those without HIV infection^1^-^5^. Compared with that of non-HIV-infected women, the infection rate of HPV in the reproductive tract of HIV-infected women is also significantly higher. This double infection further decreases the cellular and humoral immune functions, resulting in a decrease in HPV clearance ability^6^-^9^. Persistent HPV infection leads to a significant increase in the incidence of cervical epithelial precancerous lesions, which eventually leads to the occurrence and rapid progression of invasive cervical cancer^10^-^12^.

It has been found that HPV DNA exists in a free state in benign lesions caused by low-risk HPV, while most HPV DNA is integrated into the host cell DNA in cervical cancer caused by high-risk HPV, which may be one of the reasons for the high incidence of invasive cervical cancer caused by HIV combined with HPV infection^13^-^15^.

Intercellular communication is a fundamental process of multicellular organisms to complete their normal physiological functions. Intercellular communication can transmit information through direct cell contact or signal molecules secreted by cells. In the past two decades, a third way of intercellular communication has been proposed that mediates intercellular signal transduction through extracellular vesicles (EVs)^16^-^18^. Although the release of apoptotic bodies during apoptosis has been reported for a long time, the release of vesicles from cell membranes by healthy cells has been recently identified. Exosomes are extracellular vesicles surrounded by a phospholipid bilayer with a diameter of 50-100 nm that can be released or induced by resting cells. The release of exosomes is caused by the formation of a multivesicular body (MVB) by inward budding into the lumen and then by the fusion of the multivesicular body and the cell membrane^19^-^21^. Exosomes not only transports RNA, microRNAs, oncogenic receptors and HIV particles horizontally but also have antigen presenting, immune activation and immunosuppressive activities, expressing D63, CD81, CD9, LAMP1, TSG101 and other markers^22^-^26^.

MicroRNAs (microRNAs) are endogenous noncoding RNAs with regulatory functions and are found in eukaryotic organisms^27^, ^28^. They are approximately 20-25 nucleotides in size. Recent studies have shown that microRNAs are involved in a variety of regulatory pathways, including development, viral defense, hematopoietic processes, organogenesis, cell proliferation, apoptosis, fat metabolism, and so on^29^-^31^. In this study, we found that exosomes derived from HIV-1-infected cells carrying miR-155-5p could promote the malignant progression of cervical cancer cells and thus constitute pathogenic mechanisms to promote the progression of HIV-1-associated cervical cancer.

## Materials and Methods

### Cell culture and siRNA transfection

The J1.1 cell line was obtained from the NIH AIDS Reagent Program. Jurkat cells and human cervical cancer cells CaSki, Siha and HeLa were cultured in high sugar HyClone DMEM. Ten percent fetal bovine serum (Cell-Box) and 1% penicillin-streptomycin were added to the basic DMEM. All cell lines were cultured in a humidified incubator containing 5% CO_2_ at 37°C. Lipofectamine RNAiMAX was used for siRNA transfection. The sequences of the siRNA used to suppress ARID2 expression were as follows: sense 5'-GGCCGAGGAAUGGGCUCCGTT-3' and antisense 5'-CGGAGCCCAUUCCUCGGCCTT-3'. The scramble control siRNA sequences were as follows: sense 5'-UUCUGGGAACGUGACACGUTT-3' and antisense 5'-ACGUGACACGUUCGGAGAATT-3'.

### Exosome quantification and identification

Exosomes were prepared from cell supernatants by differential ultracentrifugation with filtration steps. Cell culture medium (CM) was a mixture of protease inhibitors with a concentration ratio of 1:1,000, which was derived from cells at 80% confluence with a sterile fusion degree. CM was quickly filtered with a 0.22 µm filter (Millipore) to separate intact cells and debris. Ultraspeed centrifugation at 120,000 g and 4 °C for 2 hours was performed. The vesicles were washed and resuspended in 1 mL cold PBS and then centrifuged at 120,000 g 4°C for 2 hours. Then, the pellet was resuspended in 100 ml cold PBS and transferred to a tube with low adhesion. A drop of the exosome suspension was placed on a copper net, incubated at room temperature for 5 minutes, stained with 2% uranyl acetate for 30 seconds, then dried for 60 minutes, and observed by transmission electron microscopy.

### Extraction of RNA from exosomes

After the exosomes were extracted by ultracentrifugation, 50 µl PBS was used to resuspend the exosome pellet. The samples were mixed with 700 µl pyrolysis solution, scraped, homogenized, and incubated at room temperature for 5 minutes. After incubation at room temperature for 3 min, the samples were centrifuged at 4°C for 15 min at 1.2×10^4^ g. The upper liquid phase was transferred to a new EP tube (avoiding contacting the middle layer), 1.5 times the volume of absolute ethanol was added, and the sample was mixed by pipetting. Then, 700 µl of sample was placed in the filter column of a 2 mL collection tube. The cap was tightened and the sample was centrifuged at room temperature (>8 000 g) for 15 seconds to remove the effluent. Then, 700 µl of RWT detergent was added to the filter column, the cover was tightened, and the effluent was removed after centrifugation at room temperature (>8 000 g) for 15 seconds. RPE eluent (500 µl) was added to the filter column, the cover was tightened, and the effluent was removed after centrifugation at room temperature (>8 000 g) for 15 seconds. Next, 500 µl 80% ethanol was added to the filter column, and the cap was tightened. After centrifugation at room temperature (>8 000 g) for 2 min, the effluent and collection tube were removed. Then, the filter column was placed into a new 2 mL collection tube, the cap was opened, and the sample was centrifuged at room temperature for 5 min at 1.6×10^4^ g. The membrane was dried, and the liquid and collection tube were discarded. Then, the filter column was put into a new 1.5 mL collection tube, 14 µl of enzymatic water was added to the center of the filter column membrane, the column was lightly covered, the sample was centrifuged at 1.6×10^4^ g for 1 min, and the RNA was eluted.

### RNA isolation and qRT-PCR

Total RNA was isolated using TRIzol reagent (Invitrogen) according to the manufacturer's instructions. Reverse transcription reactions were performed with the PrimeScript RT reagent Kit (TaKaRa). Quantitative PCR was performed with the SYBR Premix ExTaq Kit (TaKaRa) on a CFX96 Real-Time System (Bio-Rad) by following the manufacturer's instructions. The following primer sequences were used to detect each gene: 5'-ACTCACCTCTTCAGAACGAATTG-3' and 5'-CCATCTTTGGAAGGTTCAGGTTG-3' for IL6, 5'-TGGACCCCAAGGAAAACTGG-3' and 5'-ATTTGCTTGAAGTTTCACTGGCA-3' for IL8, 5'-CAGTGTGTCGGATTATCTGCG-3' and 5'-GCATGACGTGCTTGCTTTCATT-3' for ARID2, 5'-ACTGCGGATCTCTGTGTCAT-3' and 5'-AGAGTCCCTGCATCTCAGAGT-3' for TGFB1, 5'-AACGAGACGACGACAGACTTTTTTTTTTTTTTTV-3' for miRNA reverse transcription, universal miRNA qRT-PCR primer 5′-AACGAGACGACGACAGAC-3′, 5′-GCAAATTCGTGAAGCGTTCCATA-3′ for RNU6, and 5'-CGGGTTAATGCTAATCGTGATAGGGGTT-3' for hsa-miR-6857. PCR was performed under the following conditions: 95 °C for 15 s, 40 cycles of 95 °C for 5 s and 60 °C for 30 s. The relative expression level of each probed mRNA was then normalized to the expression level of the housekeeping gene using the 2^-∆∆CT^ method.

### Cell proliferation, migration, and invasion

Five thousand cells were added to each well of 96-well plates, and each group was repeated three times. When cells adhered to the wells, the OD value was recorded at 0 h. The OD value at 450 nm was measured for the first time. Then, it was measured five more times every 24 hours. To measure the OD value, the culture medium in the 96-well plate was first removed, 10% CCK8 solution was added to fresh culture medium, and 100 µL premixed solution was added to each well to avoid bubbles. The OD 450 nanometer value was measured after standing in an incubator at 37°C for 1 hour.

### Immunoblotting assay

Total cellular protein was extracted using RIPA lysis buffer with PMSF and phosphatase inhibitor solution. The concentration of the proteins was determined by a BCA protein quantitative kit. The protein samples (40 μg/lane) were separated by SDS-PAGE and transferred onto activated PVDF membranes (Invitrogen, CA). After blocking with 5% skim milk, the membranes were incubated with primary antibodies at 4°C overnight. The primary antibodies and dilutions were as follows: anti-ARID2 (1:1000, Cell Signaling Technology, USA), anti-CD63, anti-CD9, anti-p-ERCC5, anti-CD81, anti-p-NF-KB, and anti-NF-KB (1:500, Proteintech, USA). After washing with TBST, the membranes were incubated with the appropriate secondary antibodies. The protein bands were visualized with an ECL kit (Thermo, USA).

### High-throughput sequencing analysis

After running the sequencing program, clean data were obtained by filtering the raw reads. The clean data were aligned to the reference genome and a small RNA database by using the alignment software AASRA (Anchor Alignment-based Small RNA Annotation). AASRA is based on a new alignment algorithm, CG_anchor alignment, which adds a unique anchor sequence to both ends of the sequence and the reference sequence and then aligns them to obtain quantitative results of the expression of unique reads. Small RNAs were identified and classified by comparison with known databases such as microBase, Rfam11.0, UCSC and piRNA Bank. To assign a unique annotation to each small RNA, the small RNAs were annotated in sequence according to the priority order microRNA > piRNA > snoRNA > Rfam > other small RNA. New microRNAs were predicted using the software microDeep2. The TPM algorithm was used to standardize the expression level of small RNAs, and DEGseq software was used to screen the differentially expressed small RNAs. The differentially screened microRNAs were predicted by using miRanda, TargetScan and starBase software. GO significant enrichment analysis and KEGG pathway significant enrichment analysis were carried out with the predicted target genes of the differentially expressed microRNAs to further understand the biological functions of the genes and determine the main biochemical metabolic pathways and signal transduction pathways in which target genes of differentially expressed microRNAs participate. To determine whether there was a significant difference in the expression of microRNAs between two groups of exosome samples, the log2 ratio was used to compare the difference in the expression of microRNAs, and | log_2_ (fold change) | (> 1) was significant.

### Statistical analysis

All statistical data were analyzed by SPSS 23 statistical software. All experimental procedures in this study were repeated more than two times. Comparisons between two groups were made by Student's *t*-test, while comparisons among more than two groups were made by one-way ANOVA. Data were statistically significant when the *p* value was less than 0.05.

## Results

### Characterization of exosomes derived from HIV-infected T cells

**Fig 1:**
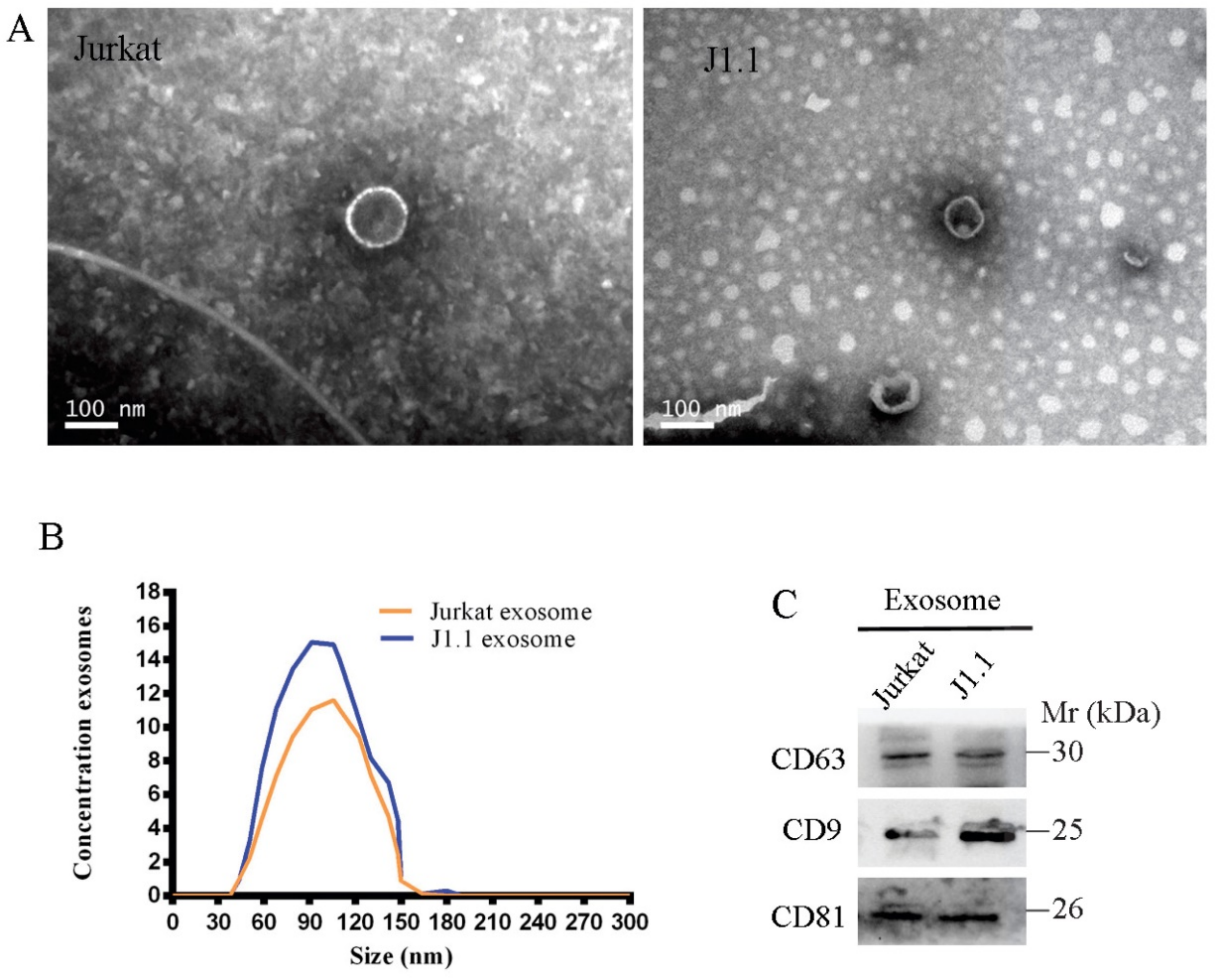
A, B Exosomes released by different cells was detected by electron microscopy and NanoSight particle tracking analysis. Scale bar, 100 nm. C Immunoblotting assay of indicated proteins in exosomes from different cell lines.

We isolated and purified exosomes from a latently HIV-1-infected human Jurkat T-cell line and the respective non-HIV control Jurkat cells by the standard exosome isolation method of ultracentrifugation at 100,000 g for 16 h at 4°C. The structure and size of the particles isolated from cell culture supernatants were determined by electron microscopy and NanoSight particle tracking analysis (Fig. 1A, B). Interestingly, we found that many more exosomes were secreted from latently HIV-1-infected cells than non-HIV control cells by quantification analysis of exosomes isolated from an equal number of cells. The exosome markers HSP70, CD63, CD9, and CD81 were further detected in exosomes from all four cell lines by immunoblotting (Fig. 1C).

### HIV-infected T-cell-derived exosomes promote cervical cancer cell proliferation, migration and invasion

**Fig 2:**
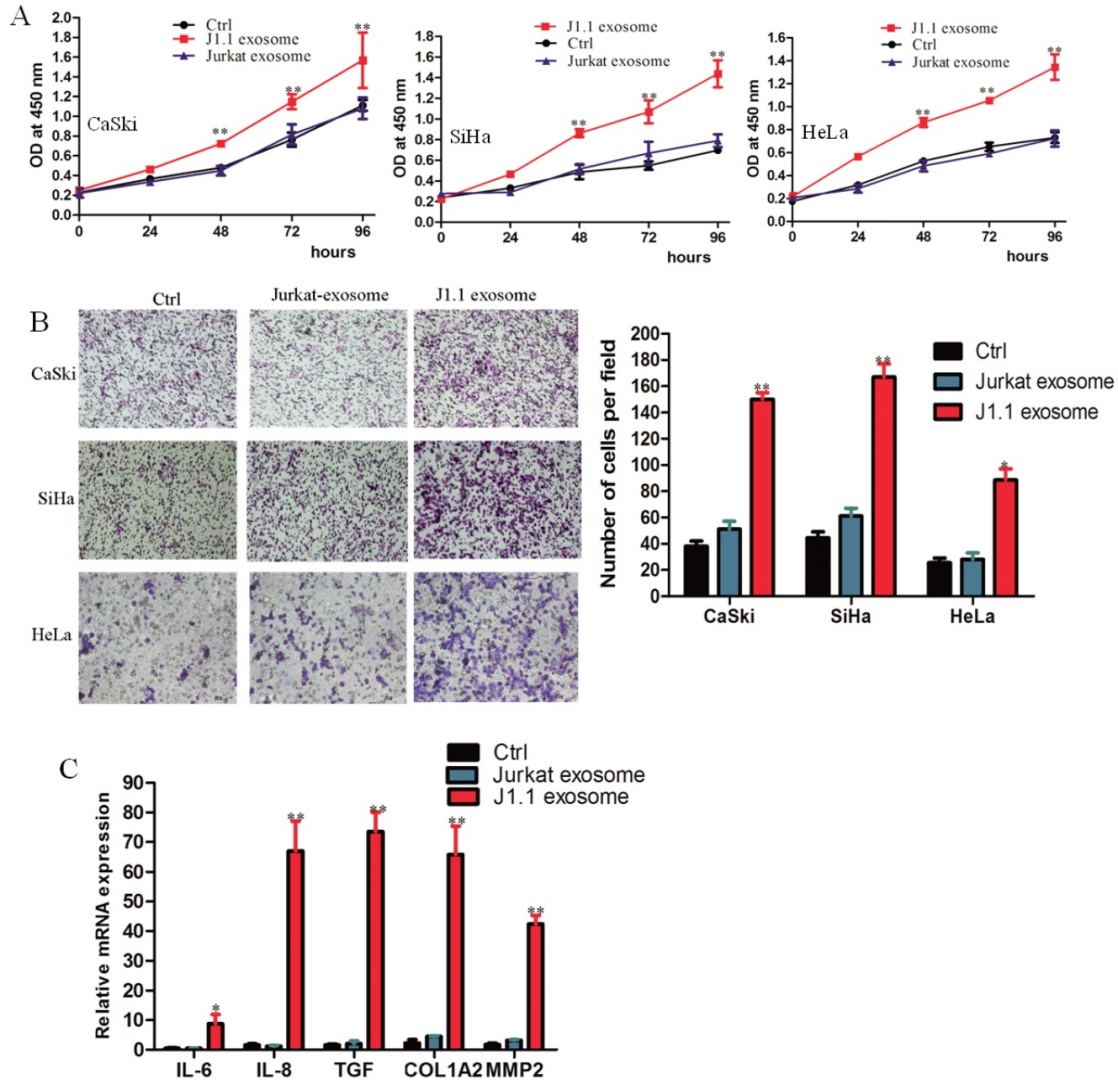
HIV-infected T-cell-derived exosomes promote cervical cancer cell proliferation and migration. (A) The effect of exosomes on the proliferation of CaSki, SiHa, and HeLa cells treated with 4 × 10^9^ exosomes/ml from J1.1 (J1.1 Exo) or Jurkat (Jurkat Exo) cells. Ctrl, serum-free medium. Error bars, ± s.d., n=3,*P < 0.05, **P < 0.01. (B) Migration assays of cervical cancer cells treated with equal quantities of exosomes derived from J1.1 cells, Jurkat cells or blank control. Migrated cells were counted, and representative images are shown. (×100) (C) IL-6, IL-8, etc. gene expression in CaSki cells treated with exosomes released by J1.1, Jurkat or control cells were detected by qRT-PCR analysis. Error bars represent three independent experiments.

To detect the transport of exosomes, we cocultured the exosomes isolated from culture supernatants of HIV-1-infected J1.1 cells or from control Jurkat cells and cervical cancer cells CaSki, SiHa, and HeLa. J1.1 cell-derived exosomes significantly promoted the proliferation and invasion of cervical cancer cells compared with control Jurkat cell-derived exosomes (Fig. 2A, B). Interestingly, cervical cancer CaSki cells cocultured with exosomes from J1.1 cells expressed higher levels of proinflammatory genes, such as IL-6, IL-8, TGF-β, collagen type I (COL1A2), and matrix metallopeptidase 2 (MMP2) (Fig. 2C), which play an important role in regulating the inflammatory microenvironment and promoting the progression of cervical cancer. In conclusion, these results demonstrate that exosomes from HIV-infected Jurkat cells contribute to the malignant progression of cervical cancer.

### MiR-155-5p in HIV-infected T-cell exosomes mediates cervical cancer progression

It has been reported that microRNAs are abundant in exosomes and play a key role in cell-to-cell communication^32^. Therefore, we hypothesized that microRNAs in HIV-infected T-cell exosomes promote the proliferation, invasion and metastasis of cervical cancer cells. To identify which miRNAs were involved, we conducted an Affymetrix multispecies miRNA-4 array to detect the miRNA expression profile of exosomes derived from J1.1 cells and Jurkat cells. A total of 474 significantly differentially expressed miRNAs were compared and are shown as heatmaps in Fig. 2A. Twenty-one of the most upregulated miRNAs (fold change > 10) in J1.1 cell-derived exosomes were subjected to validation (Fig. 2B). Only miR-155-5p mimics promoted inflammatory gene (IL-1β, IL-6, and IL-8) expression in CaSki cells (Fig. 2C). To further investigate the role of miR-155-5p, CaSki cells were stably transfected with miR-155-5p inhibitor (Supplementary Fig. 1A). As expected, the effect of miR-155-5p on cervical cancer cells was abolished by its specific inhibitor (Fig. 2D). Collectively, these findings reveal that J1.1-derived exosomal miR-1247-3p promotes the proliferation, invasion and metastasis of cervical cancer cells (Fig. 2E, F).

### MiR-155-5p in HIV-infected T-cell exosomes directly targets ARID2 in cervical cancer cells

**Fig 4:**
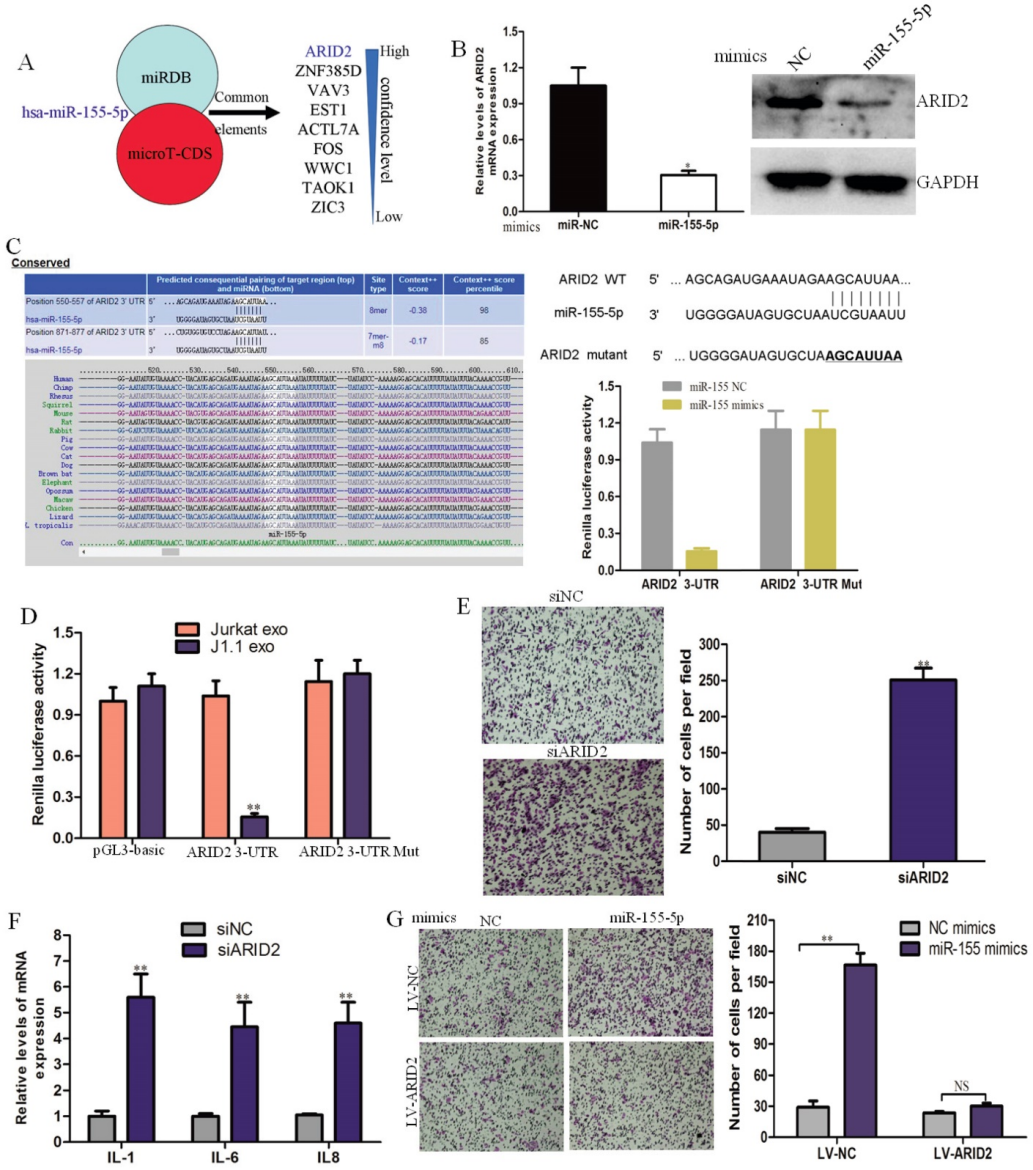
ARID2 is a direct downstream target of miR-155-5p in cervical cancer progression. (A) Target gene prediction for miR-1247-3p with two bioinformatics tools. (B) qRT-PCR and western blot assays of ARID2 expression in CaSki cells treated with miR-155-5p mimic or normal control. (C) The wild-type and a mutated type of the binding site between miR-155-5p and ARID2 3ʹ-UTR. (D) Relative luciferase activity of CaSki cells in the presence of the indicated treatments (E, F) Migration assay and qRT-PCR analysis of CaSki cells transfected with ARID2-specific siRNAs or control. (G) The effect of miR-155-5p on the migration ability of CaSki cells overexpressing ARID2.

miRDB and microTCDS (bioinformatics tools) were used to predict common target genes of exosomal miR-155-5p. The AT-rich interactive domain (ARID2)-containing family of DNA-binding proteins was verified to be a direct target of miR-155-5p and responsible for the progression of cervical cancer (Fig. 4A). ARID2 expression could be down-regulated in cervical cancer cells by miR-1247-3p at both the mRNA and protein levels (Fig. 4B). Potential binding sites of microRNA-155 were found in the ARID2 coding sequence by sequence alignment. Subsequently, we constructed dual luciferase vectors containing wild type and mutant type binding site of microRNA-155 (Fig. 4C). We found that luciferase activity decreased markedly in CaSki cells cotransfected with the wild-type binding site vector and miR-155-5p mimics. However, the luciferase activity of the mutated binding site vector was not affected by miR-155. The luciferase activity significantly decreased in CaSki cells transfected with the wild-type binding site ARID2 vector after treatment with J1.1-derived exosomes (Fig. 4D). These results reveal that ARID2 is a direct target of miR-155-5p in cervical cancer.

To identify the effect of ARID2 in cervical cancer, we knocked down ARID2 expression in CaSki cells using targeting siRNAs, and the siRNA interference efficiency was determined by qRT-PCR and immunoblotting analyses (Supplementary Fig. 1B). Migration assays and inflammatory gene expression analyses showed that silencing of ARID2 promoted cell migration and expression of inflammatory genes (Fig. 4E, F). Moreover, overexpression of ARID2 (Supplementary Fig. 1C) abolished the effect of miR-155-5p on migration (Fig. 4G). These data demonstrate that miR-155-5p directly targets ARID2 and mediates cervical cancer invasion.

### RNA-sequencing analyses revealed that the ARID2-ERCC5-NF-κB axis promotes the progression of cervical cancer

**Fig 5:**
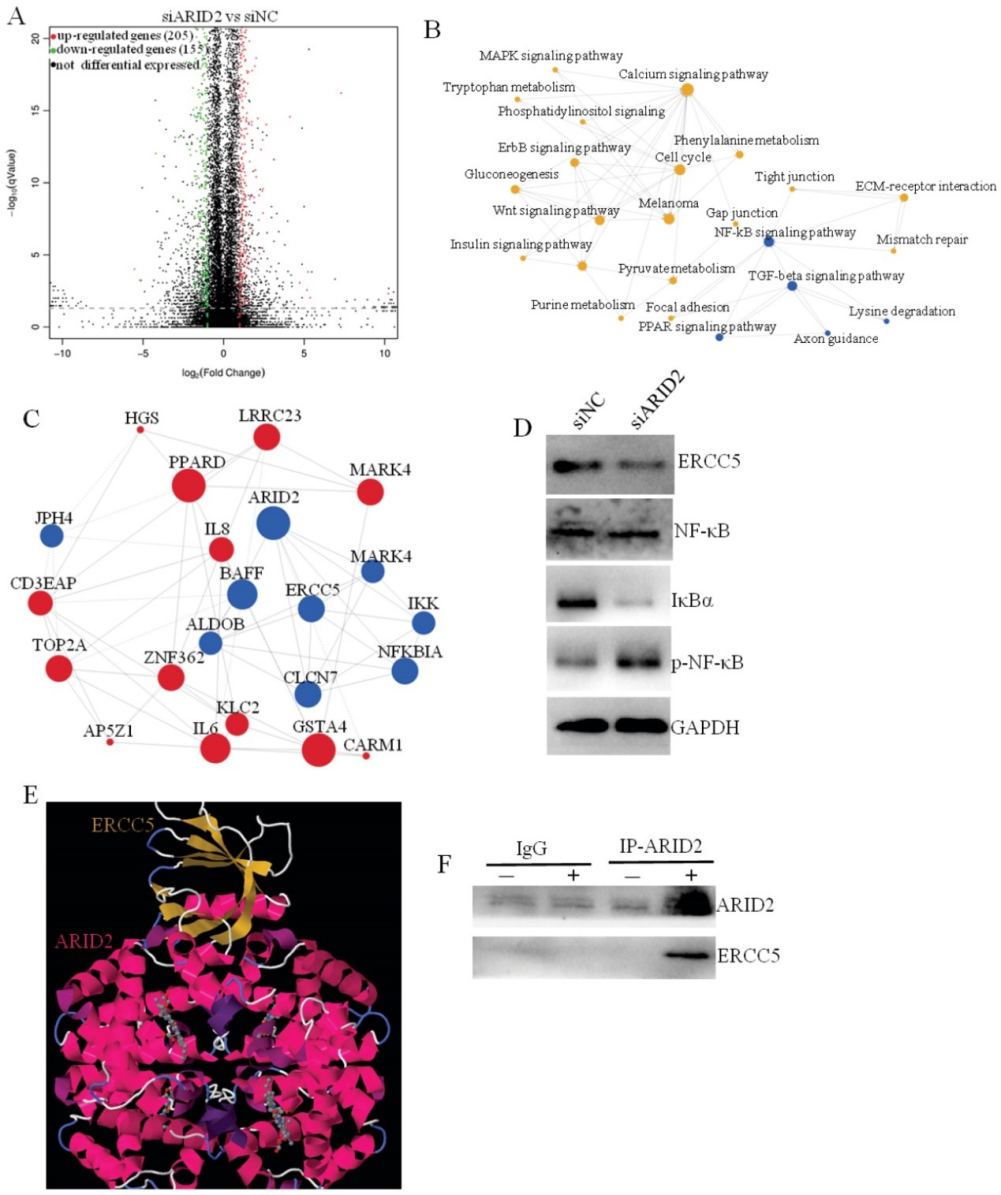
Exosomal miR-155-5p promotes cervical cancer progression via the ARID2-ERCC5-NF-κB signaling axis. (A) Volcano plot analysis of differentially expressed genes (fold change > 4; *P* < 0.05) after ARID2 knockdown. (B) Pathway network of differentially expressed genes. (C) Coexpression network of differentially expressed genes. (D) Immunoblotting assays of the indicated proteins in CaSki cells with the indicated treatments. (E, F) We used molecular docking to predict whether ARID2 and ERCC5 could bind. The predicted results show that ARID2 and ERCC5 bound by multiple potential interactions. Furthermore, we determined that ARID2 and ERCC5 bind to each other in cells by co-IP experiments.

To clarify the downstream molecular mechanism of ARID2 in promoting cervical cancer progression, RNA sequencing was used to compare differential gene expression after ARID silencing. Bioinformatics analysis revealed that there were 100 significant differentially expressed genes after ARID2 silencing, including 205 significantly upregulated and 155 downregulated genes (fold change>4) (Fig. 5A). GO and pathway enrichment analyses revealed that the differentially expressed genes were mainly involved in NF-kB signaling pathways (Fig. 5B, C). MiR-155-5p expression or ARID2 suppression also stimulated NF-κB phosphorylation and IκBα downregulation (Fig. 5D). Further analysis showed that ERCC5 was a key downstream target gene for ARID2, and the protein interaction was also verified (Fig. 5E). Furthermore, we determined that ARID2 and ERCC5 bound to each other in cells by co-IP. (Fig. 5F). In conclusion, these data demonstrate that exosomal miR-155-5p suppresses ARID2 expression via ERCC5-NF-κB signaling to promote invasion in cervical cancer.

## Discussion

A high incidence of cancer in HIV-infected people is known as HIV-associated malignancy (HAM). According to the current classification criteria by CDC, HAM can be divided into two categories: AIDS-defining cancers (ADCs) and non-AIDS-defining cancers (NADCs)^33^-^36^. ADC currently includes cervical cancer, non-Hodgkin's lymphoma and Kaposi sarcoma (KS). It was found that these ADCs were co-infected with malignant transformed cells and viruses. Because there are too many variables involved, it is difficult to establish models for relevant research^37^-^39^.

Currently, more than 80% of cancers are associated with infection. However, WHO data show that only approximately 23% of cancers are directly related to pathogenic infections. According to this, cancer occurrence must be based, at least in part, on the indirect effect of pathogen infection^40^-^43^. We can reasonably speculate that an indirect effect of infection would include at least one mechanism: the inflammatory response can be mediated by the infected cells, and then inflammatory cytokines will be enriched in the infected areas, ultimately leading to an inflammatory microenvironment conducive to the proliferation, differentiation or dedifferentiation of cancer cells.

Here, we found that HIV-1-infected T cell-derived exosomes contained miR-155-5p that promoted the proliferation, migration, and invasion of cervical cancer cells in vitro and in vivo. Most importantly, we determined that miR-155-5p was directly transferred from HIV-1-infected T cells to cervical cancer cells via exosomes and promoted invasion by decreasing the expression of its target gene ARID2 to activate the ERCC5-NF-κB signaling pathway.

Exosomal miR-155-5p derived from HIV-1-infected T cells promotes the malignant progression of cervical cancer by inducing the secretion of IL-1, IL-6 and IL-8. Among these factors, IL-6 has multiple biological effects. IL-6 is an important regulator in different kinds of chronic inflammation, but in addition to activating the immune function of the body to tumors, its main effect is to directly activate STAT3 to promote the proliferation of cancer cells and to inhibit apoptosis to help the survival of cancer cells^44^-^46^. The crosstalk between HIV-1-infected T cells and cervical cancer further elucidates the molecular mechanism of malignant progression. In conclusion, our results indicate that HIV-infected T cells can release exosomes potentially to stimulate cancer cell proliferation and establish the inflammatory tumor microenvironment, thus enhancing the growth and progression of cervical cancer.

In addition, our data demonstrate that HIV-infected T cell-derived exosomal miR-155-5p promotes the progression of cervical cancer by the ARID2-ERCC5-NF-κB signaling pathway. MiR-155-5p induces an increased secretion of IL-6 and IL-8, thereby promoting stemness, EMT, chemoresistance, and tumorigenicity of tumor cells. Our results indicate that HIV-infected patients under ART treatment contain circulating pro-tumor exosomes and that HIV-specific exosome components contribute to the tumor-promoting effect. Our study provides the underlying molecular biological mechanism for the crosstalk between HIV-infected T cells and cervical cancer, which provides a new therapeutic target for the effective prevention and treatment of AIDS-defining cancers.

## Supplementary Material

Supplementary figure.

## Figures and Tables

**Fig 3:**
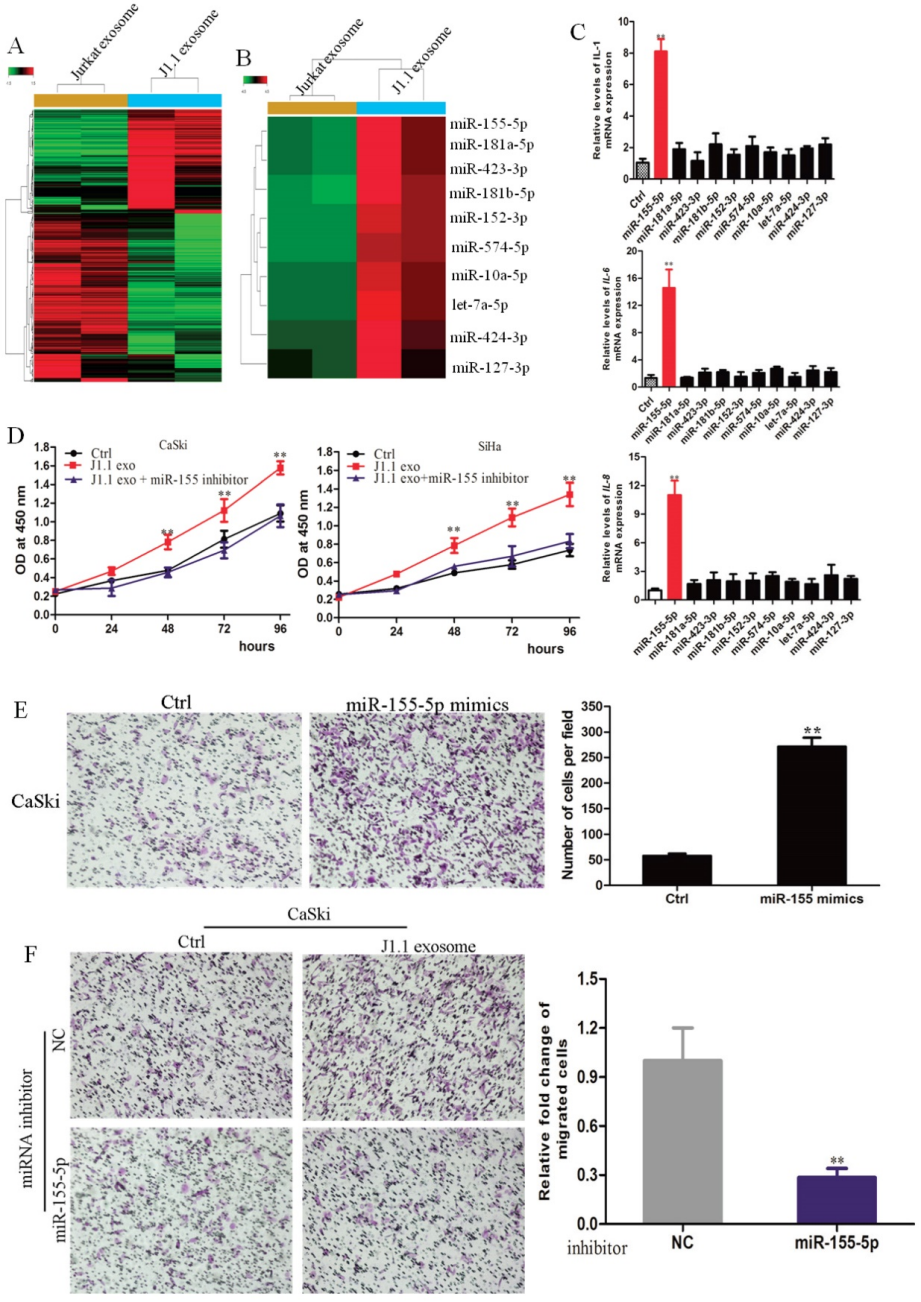
Exosomal miR-155-5p is characteristically secreted by J1.1 cells and mediates cervical cancer invasion. (A) High-throughput sequencing analysis of exosomal miRNAs from different cells is presented in a heatmap. (B) Twenty-one of the most upregulated miRNAs are presented in a heatmap. (C) qRT-PCR analysis of proinflammatory gene expression in cervical cancer cells transfected with the indicated mimics. (D) Comparison of the proliferation ability of CaSki cells treated with exosomes derived from J1.1 or control cells stably expressing miR-1247-3p inhibitor or negative control. (E) Migration assay of CaSki cells transfected with miR-155-mimic or control. Migrated cells were counted, and representative images are shown. (F) Comparison of the migration ability of CaSki cells treated with exosomes derived from J1.1 or control cells stably expressing miR-1247-3p inhibitor or negative control. Migrated cells were counted, and representative images are shown.
