# Supplementary material for: HIV-1-infected cell-derived exosomes promote the growth and progression of cervical cancer
Source: Int J Biol Sci. 2019 Sep 7;15(11):2438–47. doi: 10.7150/ijbs.38146 (PMC6775309; doi:10.7150/ijbs.38146)
Supplement: Supplementary file 1 — Supplementary figure. [file ijbsv15p2438s1.pdf]

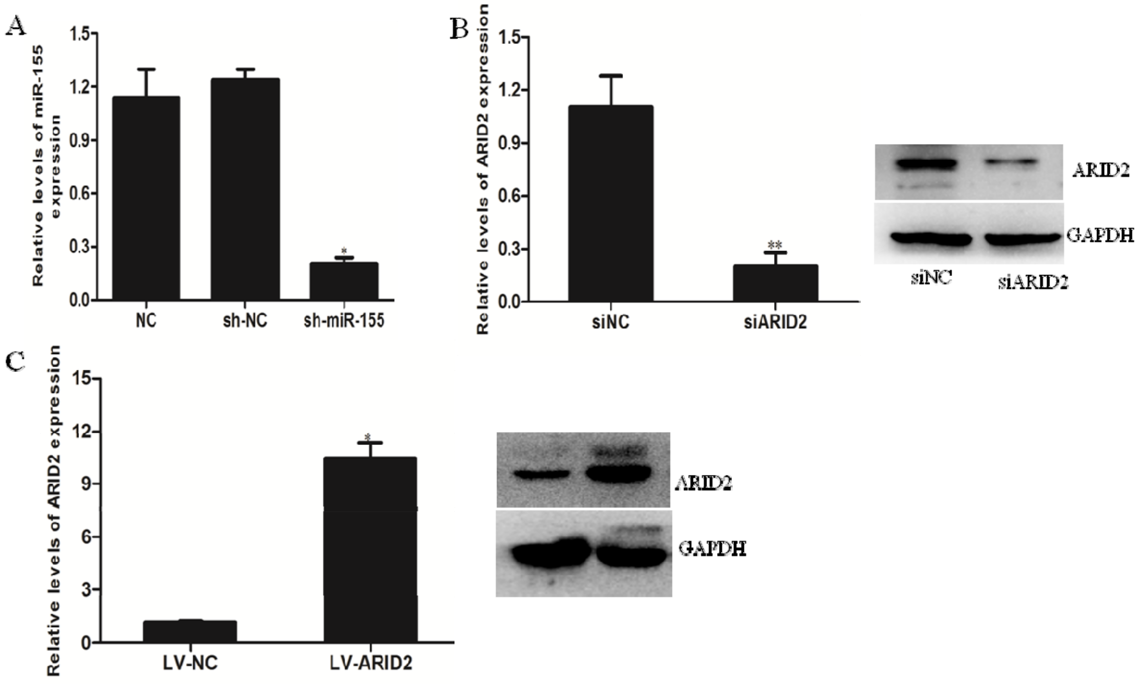

Supplementary figure (A) miR-155 expression levels was significantly knockdown in CaSki cell lines. (B) qRT-PCR and western blot was detected ARID2 expression after transfected siRNA. (C) Exogenous expression of ARID2 in CaSki cells.
